# A joint training of healthcare line managers and health and safety representatives in facilitating occupational health interventions: a feasibility study protocol for the Co-pilot project

**DOI:** 10.3389/fpsyg.2024.1340279

**Published:** 2024-05-27

**Authors:** Robert Lundmark, Alexander Agrell, Johan Simonsen Abildgaard, Jens Wahlström, Susanne Tafvelin

**Affiliations:** ^1^Department of Psychology, Umeå University, Umeå, Sweden; ^2^Department of Health, Education and Technology, Luleå University of Technology, Luleå, Sweden; ^3^Industrial Doctoral School for Research and Innovation, Umeå University, Umeå, Sweden; ^4^Department of Organization, Copenhagen Business School, Copenhagen, Denmark; ^5^The National Research Center for the Working Environment, Copenhagen, Denmark; ^6^Section of Sustainable Health, Department of Public Health and Clinical Medicine, Umeå University, Umeå, Sweden

**Keywords:** intervention, pilot study, study protocol, health care, participatory, on-job, line managers, health and safety representatives

## Abstract

Healthcare employees are experiencing poor wellbeing at an increasing rate. The healthcare workforce is exposed to challenging tasks and a high work pace, a situation that worsened during and after the COVID-19 pandemic. In turn, exposure to these high demands contributes to poor health, increased turnover, reduced job satisfaction, reduced efficacy, and reduced patient satisfaction and safety. Therefore, it is imperative that we identify measures to mitigate this crisis. One piece of this puzzle is how to implement sustainable tools and processes to improve the work environment of healthcare organizations. In this paper, we present the study protocol for the outlining and piloting of a joint training for pairs of healthcare line managers and their associated health and safety representatives in a Swedish healthcare organization. The objective of the training is to aid and advance the implementation of interventions to improve the work environment at the unit level. Following recommendations in the literature, the training is based on a stepwise approach that considers the specific context and focuses on the involvement of employees in creating interventions based on their needs. A central component of the training is the development of the pairs’ collaboration in prioritizing, developing, implementing, and evaluating the interventions. The training is based on an on-the-job train-the-trainer approach in which participants are progressively trained during four workshops in the steps of a participatory intervention process. Between these workshops, the pairs follow the same progressive steps together with their employees to develop and implement interventions at their unit. The pilot will involve four pairs (i.e., eight participants) representing different parts and functions of the organization and will be conducted over a period of three months. We will use a mixed method design to evaluate preconditions, the process, and proximal transfer and implementation outcome factors of the training. The overall aim of the pilot is to appraise its feasibility and be able to adjust the training before a potential scale-up.

## Introduction

From an employee wellbeing perspective, health care in Europe is recognized as a high-risk sector (EU-OSHA, 2023). For example, exposure to high emotional demands, a high workload, and irregular working hours increases the risk of stress and poor health among health care workers (Azzopardi-Muscat et al., 2023). In turn, these employee outcomes also affect employee turnover and sick leave and lead to lower productivity, increased costs, and adverse patient outcomes (Van Gerven et al., 2016; Niedhammer et al., 2021). In addition to this burden, at the system level, the health care sector faces several demographic challenges (Azzopardi-Muscat et al., 2023). These challenges include a shortage of employees, an aging population (i.e., with an increasing need for care), and an increasingly older workforce (i.e., with fewer employees to provide care; EU-OSHA, 2023). Rapid changes in how work is delivered also occur, with new procedures being implemented at high speed, which, for example, increases the demand for handling the introduction of a digitalized or automatized work process (Lucena et al., 2021). Furthermore, many parts of the health care sector are still struggling with the effects of the COVID-19 pandemic (EU-OSHA, 2023). A backlog of patients having to wait for treatment and health care employees in need of recovery after having been put under severe pressure and distress, contributed to an even more alarming situation (Franklin and Gkiouleka, 2021). Not surprisingly, recent studies also indicate a high prevalence of exhaustion among health care professionals (Hagqvist et al., 2022).

Though the challenges are vast one possible line of action to improve employee wellbeing is through implementing participatory workplace interventions. In this study protocol we outline the rationale for a joint training of healthcare line managers and health and safety representatives in facilitating occupational health interventions. In line with recommendations (Thabane and Lancaster, 2019), the aim of this feasibility study protocol is to increase the transparency of what will be implemented, inform the research community and the public of the project, and avoid unnecessary duplication of research and prevent selective publication and reporting of outcomes. This study protocol presents a training program to facilitate improvements of their work environment and focuses specifically on healthcare workers’ occupational health and wellbeing. Thereby we also contribute to the understanding of opportunities and pitfalls of designing and implementing health promoting practices in a healthcare setting.

### A health care contextualized participatory approach to work environment improvements

Beyond the challenges associated with contextual factors mentioned above (e.g., an aging population, high-strain work, and the COVID-19 pandemic), there are also specific conditions related to the organization of health care itself that should be considered. Health care organizations are often characterized as complex and pluralistic, with multiple objectives, and a need of flexibility in relation to unforeseen circumstances (e.g., pandemics or changes in policy; Denis et al., 2007; Burns et al., 2022). Due to the complexity of these organizations, health care constitutes a context that is particularly challenging when implementing interventions (Giusino et al., 2022). Additionally, rather than viewing healthcare work teams as the primary core of the health system, patients and their wellbeing are naturally placed in the center of operations (Anand and Bärnighausen, 2012). Patients are also the focus of legislation and guidelines surrounding processes and procedures and are often the center of attention in research (Sturgiss et al., 2022). As the link between the wellbeing of healthcare employees and patient or organizational outcomes is seldom explicitly clarified or evaluated, this relationship is often given less attention in daily operations (even though it may be crucial for the care provided; de Lange et al., 2020). Thus, although health care professionals are highly trained and skilled in detecting and treating causes and symptoms of ill health among patients, their work may sometimes come at the cost of giving lower priority to, or even neglecting, their own wellbeing.

It has been suggested that tailoring interventions to the specific local context at hand is important (von Thiele Schwarz et al., 2021). Given the complex characteristics of health care organizations, it seems particularly important to consider the challenges, structures and processes embedded in that system and culture (Giusino et al., 2022). This includes considering different organizational levels for implementing the intervention (i.e., individual, group, leader, organization, and overarching context; IGLOO; Nielsen et al., 2018) to identify and more effectively address work environment issues (De Angelis et al., 2020). Thus, rather than solely considering individual treatment, this suggests that intervention in health care organizations benefits from including a focus on employee wellbeing as a key element of the system (Teoh et al., 2023).

In fitting interventions to the specific setting of an organization, a participatory approach to designing and implementing multilevel interventions is widely recommended (von Thiele Schwarz et al., 2021). Consequently, employee involvement is also included as a core principle in intervention frameworks and can be seen as a way of further contextualizing interventions (Nielsen et al., 2021). In practice, this involves employing a bottom-up approach with employees actively participating throughout the intervention process (Schelvis et al., 2013). Accordingly, managers need to share the task of deciding what should be done, when, how, and by whom with employees or employee representatives (Lundmark et al., 2021). There are several benefits of using a participatory approach. First, it creates a better fit of the intervention with the people involved, and in turn ensures that the intervention targets the issues experienced by employees as most important to address (Randall and Nielsen, 2012). This adaptive approach also allows for aligning intervention process steps with the processes, conditions, and procedures of the specific workplace (Nielsen and Randall, 2015). Second, a participatory approach can also be seen as an empowering activity that increases employee engagement and involvement in the workplace (Lehmann et al., 2022). As such, it has been suggested that employee participation could be seen as a goal in itself, as being engaged in democratic processes at work promotes wellbeing (Abildgaard et al., 2020). Hence, recent workplace interventions in health care settings stress the importance of taking a contextualized participatory approach (e.g., Di Tecco et al., 2020; Giusino et al., 2022; Teoh et al., 2023).

### Stepwise implementation of workplace interventions

Participatory multilevel interventions are often described in a stepwise process in which employee involvement is a key feature of the entire procedure (Nielsen et al., 2010a). This stepwise process can be described in six consecutive steps (Clausen, 2022): The first step consists of *preparation,* gathering stakeholders (e.g., employee representatives, consultants, and management) to engage them in common cause and securing time and resources for change. The second step, *screening,* focuses on identifying potential areas of improvement. The identified factors include both job demands (e.g., time pressure or workload) that may increase the risk of exhaustion, and resources (e.g., role clarity or organizational support) that may reduce risk exhaustion (Bakker and Demerouti, 2017). Thus, focusing on reducing demands and/or increasing resources are viable options for interventions. As a third step, based on the results of the screening, participants *prioritize* between the potential areas of improvement, focusing on those who are perceived as either most pressing to address or most likely will bring desired improvements in the work environment. The fourth step, *action planning*, involves developing specific multilevel intervention plans to address prioritized areas. The fifth step, *implementation,* is where the planned intervention activities are put into action, with ongoing communication and follow-up activities such as adaptations to action plans. The sixth and final step encompasses process and effect *evaluation*. Evaluating allows the workplace to learn from the process and provides results that can be used to inform future interventions.

Even though all employees are ideally codrivers of this process (Nielsen et al., 2010b; Abildgaard et al., 2020), participation may not always be possible to the same degree for everyone (e.g., due to workload or working time). Designated organizational roles (and, in many cases, work environment acts) also suggest that some roles are more crucial than others regarding initiating and facilitating the intervention process (Nielsen, 2013). This may be especially true for hierarchical organizations, such as health care organizations, which have highly formalized roles, and where the hierarchical position and the mandate to initiate and implement changes are connected (Lundmark et al., 2021). In regard to safeguarding the wellbeing of employees and implementing interventions to improve their work environment, two organizational roles are often highlighted—line managers and health and safety representatives (Nielsen, 2017; Helland et al., 2021).

### Co-facilitation of interventions – a collaboration between line managers and health and safety representatives

The line manager role is the linchpin between senior management and employees, and they therefore constitute a vital communication channel between these two parties. They are often responsible for putting strategic plans into concrete action and for making prioritizations in everyday operations. They are also responsible for providing feedback and making senior management aware of when there is a lack of resources making it difficult to reach organizational objectives (Nielsen, 2017; Lundmark et al., 2020). Additionally, in the Swedish context, line managers have extensive formal responsibility for employee wellbeing at work (Frick et al., 2005). The responsibility includes ensuring continuous monitoring of work environment issues and initiating changes (i.e., interventions) to improve conditions within their work unit if needed.

The role of health and safety representatives is to safeguard the interests of employees in their unit in matters related to working conditions. This includes being part of the systematic planning, implementation, and follow-up of interventions at their workplace, functioning as a mediator who facilitates the voice and experiences of employees in this process (Hasle et al., 2019). The role of health and safety representatives is often challenging. They usually fulfill their health and safety tasks part-time and are often dependent on having a positive relationship with both their line manager and their fellow employees, as well as having a clear mandate within their organization (Rasmussen et al., 2014). Nonetheless, research on health and safety representatives has shown that when a shared understanding of a situation exists between them and their line managers, this will make intervention processes substantially more seamless and effective (Hovden et al., 2008; Helland et al., 2021).

In Sweden, and in many other European countries, health and safety representatives are appointed by the unionized employees (Menéndez et al., 2009) and their collaborative role is formalized in legislation (Steinberg, 2019). However, although this model is considered effective for creating a healthy work environment (Menéndez et al., 2009), there is often a discrepancy between legislative mandates and real-world expectations, which results in substantial variation in contributions (Helland et al., 2021). Additionally, national union reports show that in practice, health and safety representatives often lack knowledge and experience necessary for working with interventions. They also emphasize the need for further collaboration with line managers in codriving interventions (Gellerstedt, 2012; Hagström, 2021). Thus, although the importance of health and safety representatives is often highlighted, their collaboration with line managers is seldom considered or integrated as a vital part of the intervention process when designing interventions (Helland et al., 2021).

### The contextualized participatory intervention-leadership on-job training (Co-pilot) strategy

Based on this contextualized, stepwise, and collaborative approach we have developed Co-pilot. Co-pilot is a shared training for first-line healthcare managers and their associated health and safety representatives (i.e., training pairs) focusing on advancing pairwise teamwork while also training them in a systematic stepwise approach to facilitate work environment improvements at their units. The overall objective (and consequently intended outcomes) of Co-pilot is to create a positive work environment with higher levels of wellbeing and job satisfaction among healthcare workers. Thereby (indirectly) also providing conditions that may reduce turnover, increase productivity, and patient safety and satisfaction.

The Co-pilot will be implemented in two consecutive steps. First, as a pilot in which we test the feasibility of the training and subsequently as a full-scale study. To first conduct a study of training program feasibility has been recommended because this allows us to answer the “Can I/we do this?” question before testing its effects in a full-scale efficacy trial (Pearson et al., 2020). As such, feasibility studies are focused less on the effect of a program and more on how a selected group of participants perceive its content as useful, acceptable, and feasible for reaching intended outcomes (Kistin and Silverstein, 2015). The purpose being to find out whether the training provides a good fit with the needs of the participants and the organization, and to modify its content based on the participants’ appraisals. Second, based on the results of this feasibility study, necessary adaptations will be made to the content of the training, and Co-pilot will be implemented at a larger scale to evaluate its efficacy. Thereby we also investigate whether Co-pilot has the potential to become a part of integrated training programs for health care organizations. The aim of this paper is to describe the Co-pilot content and the protocol for its feasibility evaluation. Thus, elucidating (a) how this health care contextualized joint training of line managers and health and safety representatives is outlined and (b) how it will be piloted and evaluated for feasibility.

Although additional research has been called for, studies have shown that general leadership and management development programs can be beneficial for both health care employee outcomes and patient safety and satisfaction (Seidman et al., 2020). Training line managers has also been shown to enhance the effects of interventions (Nielsen et al., 2010a,b). Additionally, specific programs focused on developing healthcare line managers and specialists in implementation skills using a similar training approach indicate that on-job training in healthcare settings may contribute to training transfer (Richter et al., 2015). To the best of our knowledge this is the first joint training of line managers and health and safety representatives in a heath care context to be evaluated, the Co-pilot setup is built on the basis of the knowledge and experience gained from intervention research, as well as previous training of line managers and specialists in health care settings.

## Methods and analysis

### Study setting

This study is carried out in cooperation with a regional healthcare organization in Sweden. The healthcare system in Sweden is governed at an autonomous regional (county) level, and its services are tax funded. The 21 Swedish regional healthcare organizations are divided in three main divisions:

1. Hospital care which is provided at university and regional hospitals. Additionally, the six university hospitals provide highly specialized treatments (e.g., cancer treatments and neonatal care) for residents in regions situated in proximity.
2. Primary care units, of which some have capacity for patients to stay overnight due to the long distance from a hospital.
3. Dentistry units composed of both specialized units at the university hospital and general dental care clinics.

In total, approximately 300,000 individuals are employed by the 21 Swedish regional healthcare organizations. Beyond that, several private healthcare and dentistry companies are contracted by the regions to provide healthcare services.

### Training development

The development of the training departed from the six-step process developed by the Danish National Research Centre for the Working Environment (NFA; Clausen, 2022). Based on the guiding principles of a contextualized, stepwise, and collaborative approach, the content of the training was elaborated in two half-day workshops. This work was performed together with representatives of the participating regional health care organizations HR department and its integrated occupational health services. These representatives were chosen on the basis that they have good insight into the organization’s strategies and current work to safeguard employee wellbeing. They also have vast experience with efforts to implement interventions at different levels in the organization (both successful and unsuccessful). Another reason for involving these representatives is that they play a potentially important role in sustaining the Co-pilot initiative should it be integrated as part of the organization’s training after the study is completed.

**Figure 1:**
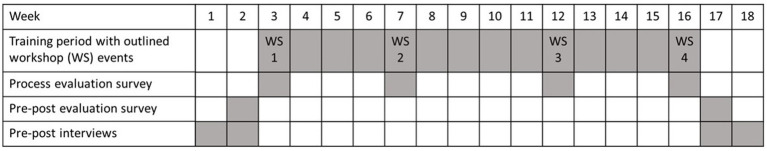
Evaluation model of the training.

As a result of these workshops, material, and tools from the NFA were adapted, and the training was developed to fit the context at hand (e.g., a healthcare setting and a joint training of line managers and health and safety representatives). As part of the planning, we strived for striking a balance between the need for sufficient time for training and the organization’s possibility to find replacement (or in other ways compensating for the perceived risks associated with having the participants removed from their units). Based on this, the training is planned to span over three months and include four consultant led workshops (i.e., 4–5 weeks apart). In this development of an integrated program logic (von Thiele Schwarz et al., 2016), we also outlined a plan for the evaluation of the training (see Figure 1).

Thus, we aimed for a comprehensive training course combining our different areas of knowledge and expertise (e.g., on current occupational health strategies and support in the organization, as well as on research-based methods and material). The purpose being to design a course that encompasses effective methods for improving employee wellbeing as well as information on how these “new” practices can be integrated with the processes and procedures of the organization. In addition, a stakeholder reference group consisting of representatives from management and unions (i.e., healthcare workers with a partially reimbursed mission and mandate to represent the employees of the organization) at the regional health care organization was established for the project. The reference group will be consulted for input and help with the recruitment of suitable participants. The group will also advise on the timing, content and data collection related to the training as well as the interpretation of the results of the training. Continuous meetings with the reference group are planned throughout the project (approximately twice a year).

### Content of the training workshops

**Figure 2:**
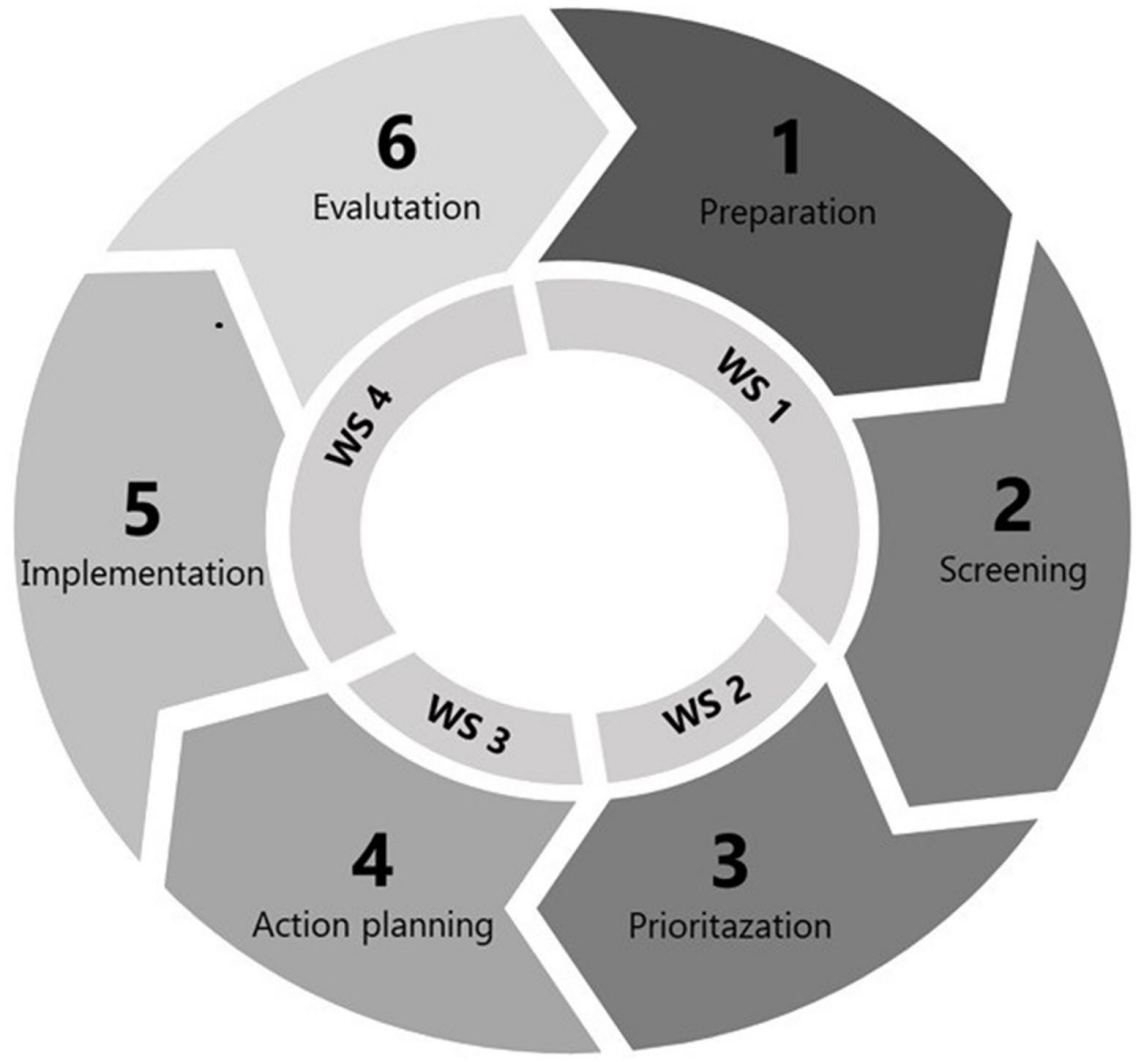
Outline of workshops in relation to the intervention process.

Each workshop will be concentrated on one or two steps of the intervention cycle (see Figure 2). The themes and the material of the workshops are first introduced in short lectures, followed by exercises. Between each workshop, the training pairs involve employees at their workplace (i.e., unit) and work together with them to complete each step of the intervention process in accordance with the methods and material that was presented at the preceding workshop. Thus, the Co-pilot applies a train-the-trainer approach (Pearce et al., 2012), in which the training pairs first practice (under guidance) each step using the methods and material introduced during workshops and then implement, step-by step, what they have learned at their unit. Consequently, the training pairs are taught how to jointly facilitate a participatory intervention process at their unit.

Workshop 1 (see also Table 1) focuses on introducing the training and stressing the importance of a sound work environment in healthcare. An exercise to enhance collaboration between line managers and health and safety representatives in facilitating interventions is carried out. Thereafter, methods of screening are introduced (i.e., using the annual work environment survey, patient evaluations, and a visual mapping tool), and the participants trained in using these methods. In workshop 2, the training focuses on identifying, prioritizing, and specifying areas for improvement, and in workshop 3, the focus is on the development of action plans. Workshop 4 targets the implementation and sustainment of action plans, as well as developing a plan for evaluation. At workshop 4, the integration of action plans into the digital follow-up system of the organizations is also introduced and practiced. Workshops 2–4 start with collective follow-up and problem solving based on the work done between the workshops. At the end of each workshop, the participants will complete a short survey in which they anonymously appraise the quality of the workshop. In addition to using NFA process tools for visual mapping, prioritizing and action planning (Wåhlin-Jacobsen et al., 2017; Clausen, 2022), the collaboration exercise is based on material from a web-based tool for enhancing line managers’ and health and safety representatives’ cooperation (Suntarbetsliv, 2023). Furthermore, we will also include material from the behavior change wheel (Michie et al., 2014) to facilitate the identification, prioritization, and specification of activities.

**Table 1:** Content of the Co-pilot training.

WS 1	WS 2	WS 3	WS 4
Work environment and health in healthcare and collaboration	Identify, prioritize, and specify intervention activities	Goal oriented action planning	Implementation, evaluation, and sustainability
6 h	3 h	3 h	3 h
Introduction to the program. Work environment and health in healthcare. Work environment mapping. Collaboration.* Mapping tool.* Introduction to between WS mapping co-facilitation assignment. WS evaluation.	*Follow-up on assignment*.* Transition of work environment mapping to identified, prioritized and specified intervention activities. Identification, prioritization and specification tool.* Introduction to between WS intervention activities co-facilitation assignment. WS evaluation.	*Follow-up on assignment*.* Goal orientation Introduction to Action planning Action planning.* Peer-feedback on action plans. * Introduction to between WS action plan co-facilitation assignment. WS evaluation.	*Follow-up on assignment*.* Implementing, evaluating and sustaining interventions. Plan for follow-up with employees.* Introduction to the organizational digital infrastructure for evaluation. Transfer of action plans to the organizational digital infrastructure for evaluation* WS evaluation.

WS, Workshop.

^*^ Participatory exercises.

### Recruitment process and participants

The recruitment of participants (i.e., training pairs) for the pilot will be conducted in collaboration with the reference group. We will aim for a mixed group of participants for the pilot, representing the variety of experience from the health care organization. We will also aim for a group of participants with different backgrounds to indicate whether the training provides an equal fit independent of such factors. In total, we will recruit four pairs (i.e., eight participants) for the pilot. Ideally, the pairs will thus represent the different divisions of the organization (e.g., from intensive care, primary care, and rehabilitation), include a variety of different healthcare occupations (e.g., nurses, medical doctors, physiotherapists), and include both male and female participants of different ages and lengths of employment (i.e., tenure). Thus, using stratified rather than random selection, the aim is to include participants who reflect the variety of the organization and its members. Using data from only eight participants from four units will not cover all aspects of the organization. However, as the focus is on evaluating the feasibility of the training, we want to establish a good working relationship and room for the participants to express their views and provide us and each other with feedback, in line with a psychologically safe climate (Edmondson et al., 2016). Therefore, we judge eight participants as an appropriate trade-off between including participants with a variety of backgrounds from different parts of the organization and being able to develop a psychologically open and safe climate. The pilot will be led by two of the researchers with the assistance of two experienced internal consultants from the organization. By cooperating with consultants, we also transfer knowledge and experience for the potential larger-scale implementation of the training in which they will take the lead role.

### Evaluation procedures

Even though the nonrandomized design of our pilot study does not fully fall within the standards of the CONSORT extension for feasibility studies, we will (as suggested; Eldridge et al., 2016) adhere to these principles when applicable for reporting our results. The feasibility study will focus on the participating line managers and health and safety representatives. It will be based on a mixed methods approach and, as such, will comprise both quantitative and qualitative elements—before, during and after its implementation—in line with recommendations for intervention, and feasibility, study evaluations (Pearson et al., 2020; Nielsen et al., 2023; see also Table 2). Before implementation, participants will be invited to answer a short digital questionnaire with questions focusing on their preconditions (e.g., readiness, motivation, and knowledge). We will also ask the participants to appraise each of the 4 workshops for content and quality of delivery by answering a short digital survey (i.e., using their smartphones). After the last workshop, participants will again answer a digital survey including the pre-implementation measures as well as measures of acceptability, appropriateness, feasibility, integration, and utility of the training. The participants will be provided with login codes to the digital surveys so that the data collection can be performed anonymously. Furthermore, semi-structured interviews with all 8 participants will be conducted before and after the implementation of the training. These interviews focused on expanding and enriching the gathered information (i.e., on participants’ perceptions of the training and the conditions surrounding the training) and thus provided a complement to the quantitative data collection [i.e., as recommended by Nielsen et al. (2021)].

**Table 2:** Constructs in the evaluations.

Construct	Origin of scale	Number of items and type of scale	Time of measure (see Figure 1)
*Pre-post evaluation*			
Outcome expectancy/realization	Fridrich et al. (2016)	2 items 5-point Likert scale	Pre-post
Readiness	Randall et al. (2009)	4 items 5-point Likert scale	Pre-post
Situational motivation	Guay et al. (2000)	9 items 5-point Likert scale	Pre-post
Direction	von Thiele Schwarz et al. (2016)	2 items 5-point Likert scale	Pre-post
Support	von Thiele Schwarz et al. (2016)	2 items 5-point Likert scale	Pre-post
Implementation climate	Jacobs et al. (2014)	3 items 5-point Likert scale	Pre-post
Pre-knowledge	Richter et al. (2015)	5 items 5-point Likert scale	Pre-post
Self-efficacy	Rigotti et al. (2008)	6 items 5-point Likert scale	Pre-post
Collaboration	Wong et al. (2009)	4 items 5-point Likert scale	Pre-post
Acceptability	Weiner et al. (2017)	4 items 5-point Likert scale	Post
Appropriateness	Weiner et al. (2017)	4 items 5-point Likert scale	Post
Feasibility	Weiner et al. (2017)	4 items 5-point Likert scale	Post
Integration	von Thiele Schwarz et al. (2016)	2 items 5-point Likert scale	Post
Utility	Adapted from: Grohmann and Kauffeld (2013)	1 item 5-point Likert scale	Post
*Process evaluation*			
Workshop appraisal	Fridrich et al. (2020)	9 items 7-point Likert scale	WS: 1, 2, 3, 4
Workshop evaluation	Developed by authors, inspired by: Richter et al. (2015)	8 items 5-point Likert scale	WS: 1, 2, 3, 4
Content of workshops	Developed by authors, inspired by Richter et al. (2015)	3–4 items (depending on workshop) 5-point Likert scale	WS: 1, 2, 3, 4
Participation frequency	Von Thiele Schwarz et al. (2016)	Attendance at workshops yes/no	WS: 1, 2, 3, 4

### Data analysis

The statistical data analysis from the quantitative data collection will be performed by comparing pre- and post-ratings (using comparative statistics such as t tests) to determine whether any differences in perceptions of these proximal outcomes can be detected. These tests can also provide information on the validity of these measures as part of the training evaluation model. We will also perform analyses of the process evaluation (i.e., workshop appraisal and evaluation measures) on an item-level basis to determine whether there are any parts of the training that are perceived as less relevant. Also, to evaluate whether there is congruence in these perceptions or if they can be seen as related to other factors (e.g., demographic or preconditional, such as job-demands and resources on a unit-level as presented in the annual employee survey). Finally, we will use the postimplementation ratings, together with the results of the interviews, to appraise the overall feasibility of the training. The pre- and post-pilot interviews will be analyzed using an exploratory thematic analysis approach, focusing on the experiences and opinions related to the Co-pilot process (Boyatzis, 1998; Braun and Clarke, 2012; Saldaña, 2021). These analyses will contribute to a more in-depth understanding of the participants’ perceptions of their preconditions, expectations and factors potentially contributing to and hindering the transfer of the training to knowledge and actions. The overall results will then be used to make relevant changes to the Co-pilot protocol before implementing the training on a larger scale.

### Ethics

All participation in the training will be voluntary, as will participation in the evaluation of the training. Written informed consent to participate in the training and in this research will be obtained from all participants. Ethical approval for this study was granted by the Swedish National Ethical Review Authority [2021–03877].

## Discussion

This pilot study will be the first, to our knowledge, to implement a contextualized joint training of health care line managers and their associated health and safety representatives with the aim of improving healthcare employees’ wellbeing. Considering the current conditions for healthcare employees (EU-OSHA, 2023) and the calls for interventions at different levels (De Angelis et al., 2020), its main contribution lies in its potential to add to the solutions available for improving the work environment and health in healthcare. The Co-pilot also contributes by its emphasis on joint training in which healthcare line managers and health and safety representatives share responsibility for supporting the implementation of the process in their unit. By promoting a shared understanding and clarification of these two roles in promoting employee wellbeing, the training focuses on collaboration as an important mechanism for the successful implementation of interventions. Although the collaboration between mangers and health and safety representatives is in center of work environment improvement guidelines (Frick et al., 2005), it is currently an under-studied phenomenon (Helland et al., 2021). The Co-pilot’s emphasis on this collaboration will therefore also contribute to clarifying the potentials and pitfalls of healthcare line managers’ and health and safety representatives’ joint efforts to facilitate improvements of the work environment. The training uses an on-job train-the-trainer method in which the participants depart from their own real-world cases as part of the training. It also focuses on flexibility and integration into practice by stressing a bottom-up approach. Thereby, identification of the targets for interventions, and implementing and evaluating these interventions is addressed locally, and based on the perceived needs of the units’ employees. Stressing the collaboration between line managers and health and safety representatives in this training potentially also adds to the expansions of factors to consider in multi-level interventions models and frameworks. Currently, in these models and frameworks the collaborative work between these stakeholders is often given sparse attention, even though it has shown to be paramount for reaching intended outcomes (Helland et al., 2021).

The pilot will hopefully provide useful information on the feasibility of the Co-pilot endeavor and information on aspects that need adaptation and hence help produce an efficient training to be implemented at a larger scale. In addition, the interventions that are produced in the pilot may also be used as examples in the training material in future large-scale implementation of the Co-pilot. Thus, by observing, documenting, and sharing these examples with the reference group and with future participants, we will hopefully also contribute to overall learning in the organization. From an organizational perspective, it is highly likely that the needs for change are shared between similar work units or that common prerequisites required for facilitating change can be addressed at higher organizational levels. By observing the work done in the training, we therefore also aim to contribute to identifying such communalities and helping the organization integrate these experiences in their operational planning at higher managerial levels.

### Limitations

As with all research, this study has several limitations. First, in the development of the training, the collaboration did not directly include healthcare employees. Not including them as part of these workshops could be seen as lowering ambitions in regard to involvement. Instead, the collaboration at this stage focused on involving a small group of representatives with knowledge and insights into previous efforts as well solid knowledge of “what works” in this context. The HR and occupational health service representative’s role in the organization is mainly to develop and implement strategies and facilitate interventions. Thus, a primary reason for involving them was to ensure that the training could be well integrated into current strategies and practices. We also view them as important stakeholders in sustaining the “training” within the organization after the Co-pilot project has ended. Additionally, we view the feasibility study as part of the Co-pilot development; therefore, the appraisals and suggestions for changes from the participants can be seen as a second step in this collaborative process.

Second, the planned nonrandomized selection of participants for the pilot may result in a sample that does not reflect the targeted population sufficiently. Participants potentially have greater readiness for change, an already sound work environment and good collaboration within the pairs. Alternatively, they may choose to sign up because of a very problematic situation for which they seek a solution. Although participants will be recruited from different operations, given that we only seek four pairs for the pilot, there is a risk that the selection of these participants is biased. There is also a risk that even if training pairs are highly motivated, employees may be less intrigued by allocating time and energy to participate in the process. However, we will collect data on preconditions, as well as process data during training, which can help indicate if this is the case. Cautiousness will guide interpretations of the feasibility results, and we will consider the risks of these biases when planning for the future large-scale design of training. The use of only eight participants also restricts the potential for conducting advanced statistical analyses and for drawing conclusions about pre-post changes in perceptions. However, as the overall aim of the pilot is to evaluate its feasibility, our main objective is to investigate participants’ appraisal of the training content and perceptions of the process (e.g., feasibility, acceptability, appropriateness, and utility) to improve its outlining before scale-up. Restricting the number of participants to eight, we therefore believe is a reasonable trade-off in relation to establishing a psychologically safe climate, and not putting too much burden on the organization.

A third potential limitation, or risk, is the timing of the training. Different units have different planning and so the timing of when the training takes place may affect participants differently (e.g., higher or lower workloads during implementation). This may influence the possibilities for participation and thus exposure to the training, both for the participants in the training and the employees at their units. Additionally, the planned dissemination of training workshops over three months may lead to unforeseen contextual factors influencing the possibility of enduring participation over time. Although there is no guarantee of success, we plan for the timing of the intervention in cooperation with the organization and follow-up on the participants’ participation in the workshops and potential hindrances for the participation of their associated employees between the training workshops. Given that the focus here is to pilot the training, we view these implementation outcomes as important inputs to the training design and will consider these results before scaling up the training. An alternative to the current design would be to allocate pairs to different trainings in time to fit with their individual unit needs. Even though a fixed timing entails a risk of adding to the burden for some, we believe that a contribution to the training is the mixed training group, in which participants has the potential to acknowledge and learn from each other’s challenges.

## Data availability statement

The raw data supporting the conclusions of this article will be made available by the authors, without undue reservation.

## Ethics statement

The studies involving humans were approved by Swedish national ethical review authority. The studies were conducted in accordance with the local legislation and institutional requirements. The participants provided their written informed consent to participate in this study.

## Author contributions

RL: Conceptualization, Funding acquisition, Methodology, Supervision, Writing – original draft. AA: Conceptualization, Methodology, Project administration, Writing – review & editing. JA: Conceptualization, Funding acquisition, Methodology, Supervision, Writing – review & editing. JW: Conceptualization, Funding acquisition, Supervision, Writing – review & editing. ST: Conceptualization, Funding acquisition, Methodology, Supervision, Writing – review & editing.
